# Targeting JAK/STAT Signaling Attenuates Postoperative Recurrence in an HLA-B27 Transgenic Rat Model of Crohn’s Disease

**DOI:** 10.3390/ijms27219487

**Published:** 2026-10-24

**Authors:** Aurélie Blondeaux, Caroline Valibouze, Marie Titécat, Jean Guillaume Letarouilly, Caroline Dubuquoy, Matthias Danes, Laurent Dubuquoy, Philippe Zerbib, Benoît Foligné, Pierre Desreumaux, Silvia Speca

**Affiliations:** 1 U1286 INFINITE (Institute for Translational Research in Inflammation), Inserm, CHU Lille, Université de Lille, 59000 Lille, France; aurelie.blondeaux59@gmail.com (A.B.); caroline_valibouze@hotmail.fr (C.V.); marie.titecat@univ-lille.fr (M.T.); jeanguillaume.letarouilly@chu-lille.fr (J.G.L.); matthias.danes@univ-lille.fr (M.D.); laurent.dubuquoy@inserm.fr (L.D.); philippe.zerbib@chu-lille.fr (P.Z.); benoit.foligne@univ-lille.fr (B.F.); 2 Department of Hepato-Gastroenterology, CHU Lille, 59000 Lille, France; 3 Department of Digestive Surgery and Transplantation, CHU Lille, 59000 Lille, France; 4 Department of Rheumatology, FHU PRECISE, CHU Lille, Université de Lille, 59000 Lille, France; 5 Intestinal Biotech Development, 59045 Lille, France; carodubu@yahoo.fr

**Keywords:** Crohn’s disease (CD), post-operative recurrence (POR), HLA-B27 transgenic rats, endoscopy, JAK/STAT signaling, Tofacitinib

## Abstract

Postoperative recurrence (POR) remains a common complication following ileocecal resection (ICR) in patients with Crohn’s disease (CD), and no therapy is currently approved. This study aimed to study the benefits of JAK/STAT pathway inhibition in preventing POR using an ICR model in HLA-B27 transgenic (Tg) rats. Twenty-nine Tg rats underwent ICR at 12 weeks of age (W12). TOFA (15 mg/Kg/day) or placebo was administered at W10 as a preventive intervention. Disease extent and severity were assessed by monitoring body weight, stool consistency, performing endoscopy (W15) and evaluating macroscopic and histologic scores (W18). Ileal STATs phosphorylation was assessed by Western blotting and immunohistochemistry, while inflammatory cytokine expression was quantified by qRT-PCR and ELISA. In the placebo-treated Tg group, 57% of rats exhibited mucosal ulcerations in at least 20% of the anastomotic area (macroscopic score ≥2), which correlated with an endoscopic score ≥9. In contrast, only 13% of TOFA-treated Tg rats had a macroscopic score ≥2 and all had endoscopic scores ≤9. TOFA reduced mucosal inflammation and decreased ilealSTAT3 phosphorylation and pro-inflammatory cytokine expression. These findings provide a preclinical rationale for further investigating JAK inhibition as a potential preventive strategy against POR in CD.

## 1. Introduction

Despite advances in therapeutics and disease monitoring in Crohn’s disease (CD), 80% of patients still require surgery during their lifetime [1,2]. However, surgery is not curative and, in the case of terminal ileal resection, is associated with a high risk of postoperative recurrence (POR). In the seminal prospective cohort by Rutgeerts et al. in 1999, endoscopic recurrence was observed in 73% of patients within one year after ileal resection (ICR), although only 20% had clinical symptoms at that time [3,4]. In France, two prospective nationwide cohorts, the REMIND and GETAID cohorts, reported endoscopic POR in 47% and 57.6% of patients, respectively, within one year after ileocolonic. In the GETAID cohort, clinical recurrence was also observed in 11.3% of patients during the first year after surgery. More broadly, in the absence of postoperative preventive treatment, endoscopic POR has been reported to affect approximately three-quarters of patients within the first year and more than 85–90% over the long term, whereas clinical POR may occur in approximately 50% of patients within 5 years after resection [5,6].

The pathophysiological mechanisms driving POR in CD are complex and multifactorial. Abnormal interactions between the mucosal/mesenteric immune system and the intestinal microbiota, promoted by surgical techniques and environmental factors, are pivotal hallmarks in POR dynamics [7]. Presently, two main strategies are proposed to prevent POR: i. endoscopy-guided pharmacologic therapy, where ileocolonoscopy at 6–12 months post-surgery guides therapeutic decisions according to the presence and severity of endoscopic recurrence [8], and ii. early post-operative prophylaxis, typically initiated 4–6 weeks after surgery and with agents targeting tumor necrosis factor α (TNFα) or leukocyte α4β7 integrins [9,10]. Recently, ileal transcriptome analyses of CD patients identified a POR-associated gene signature characterized by upregulation of interleukin (IL)-23 and IL-17 and abnormal Janus Kinases (JAK)/Signal Transducers and Activators of Transcription (STAT) pathway activation [11].

The JAK family is composed of 4 phosphotransferases (JAK1, JAK2, JAK3 and TYK2) associated with the cytoplasmic domain of the type I/II cytokine receptors, which mediate phosphorylation and subsequent activation/nuclear translocation of specific STAT family members [12]. The primary function of this pathway is to regulate signal transduction of several cytokines and to drive polarization toward Th1, Th2, Th9 and Th17 profiles [13]. Tofacitinib, filgotinib and Upadacitinib are the three JAK inhibitors approved by the European Medicines Agency and/or the American Food and Drug Administration for managing patients with IBD [14]. Tofacitinib, a pan-JAK inhibitor with preferential selectivity for JAK1 and JAK3, was the first small-molecule drug approved for the treatment of moderate-to-severe ulcerative colitis (UC) in 2018 [15,16]. Filgotinib, a selective JAK1 inhibitor, was subsequently approved for UC in the European Union in 2021, followed by Upadacitinib, another selective JAK1 inhibitor, which received approval for treating UC in 2022 and CD in 2023 [17,18]. Despite these advances, the potential of JAK inhibition in preventing POR in CD remains unexplored. Importantly, as JAK1, JAK2 and JAK3 orchestrate STAT3 phosphorylation, a central pathway in POR pathogenesis [11], we hypothesized that blocking this axis with a pan-JAK inhibitor, such as tofacitinib, could improve disease recurrence. Thus, we developed a robust and highly reproducible model of ileocecal resection (ICR) and end-to-end anastomosis in HLA-B27 transgenic (Tg) rats, which faithfully recapitulates CD-associated POR and provides a valuable platform for the discovery of novel therapeutic strategies.

## 2. Results

### 2.1. Clinical Parameters

No mortality was observed in Tg rats receiving PBS or Tofacitinib (TOFA) daily by oral gavage. We weekly assessed the evolution and severity of colitis by monitoring body weight, fecal blood and stool consistency. Both groups showed an increase in body weight from baseline (W10) to ileocecal resection (ICR) (W12). PBS-treated rats showed a significant transient body weight loss one week after ICR (W13) (104, IQR: 100.8–108.3 at W12 vs. 96.5, IQR: 92.75–102 at W13; *p* = 0.015), followed by gradual recovery until sacrifice. Tg rats receiving TOFA showed a similar overall pattern of weight changes, but without significant variation between W12 and W13 (Figure 1a). No bloody stools were observed in Tg rats; thus, the DAI score mainly reflected weight loss and stool consistency. At baseline (W10), 34% of all Tg rats (5 rats per group) had liquid stools (referable to a score of 2). At W13, PBS-receiving rats showed reduced stool consistency with a global increase in median DAI score, rising to 2.5 (IQR: 1–3), compared with 1 (IQR: 1–2) at W12 (*p* = 0.052). TOFA prevented the loss of stool consistency, maintaining stable DAI scores throughout the treatment, though without a significant difference compared to controls (Figure 1b).

**Figure 1:**
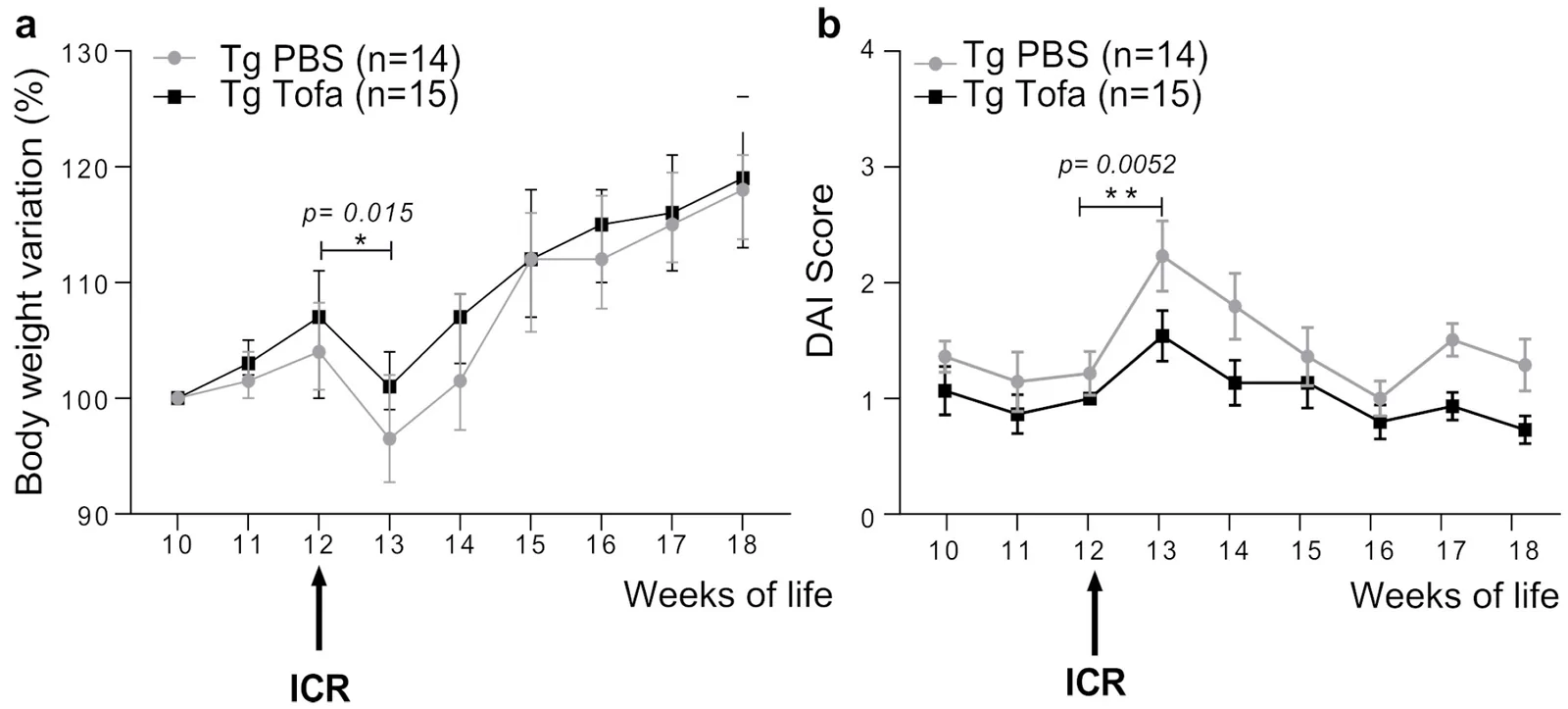
Body weight and DAI score evolution. (**a**) Weekly monitoring of body weight evolution compared to W10 for Tg PBS (*n* = 14) and Tg TOFA (*n* = 15) rats. (**b**) Weekly scoring of DAI evolution starting from W10 to W18 for Tg PBS (*n* = 14) and Tg TOFA (*n* = 15) rats. Data are quoted as median with IQR and analyzed with a Two-way ANOVA with Tukey’s correction for multiple pairwise comparisons (* = *p* < 0.05; ** = *p* < 0.01) and Sidak’s correction to compare the Tg PBS group to the Tg TOFA group.

### 2.2. Macroscopic Anastomotic Lesions and POR at W18

At W12, no gastrointestinal lesions were observed in any rats. By W18, over 90% (13/14) untreated Tg rats developed macroscopic anastomotic lesions, including: i. edema (scored 1, 4/14); ii. ulcerations without stenosis (score 2, 5/14; score 3 if >20% of the anastomotic area, 1/14); iii stenosis (score 4, 2/14). Ulceration and/or stenosis were considered markers of disease recurrence; thus, clinical POR was represented by a macroscopic score ≥2 and occurred in 57% ± 14% of untreated rats. In contrast, most animals treated with TOFA had normal macroscopic anastomosis (11/15). Compared to untreated rats, TOFA significantly reduced both the macroscopic score (0, IQR: 0–1 vs. 2, IQR: 1–2.25; *p* = 0.0002) and POR incidence (13% ± 9% vs. 57% ± 14, *p* = 0.02, odds ratio: 8.667 with 95% CI: 1.493 to 46.15) (Figure 2).

**Figure 2:**
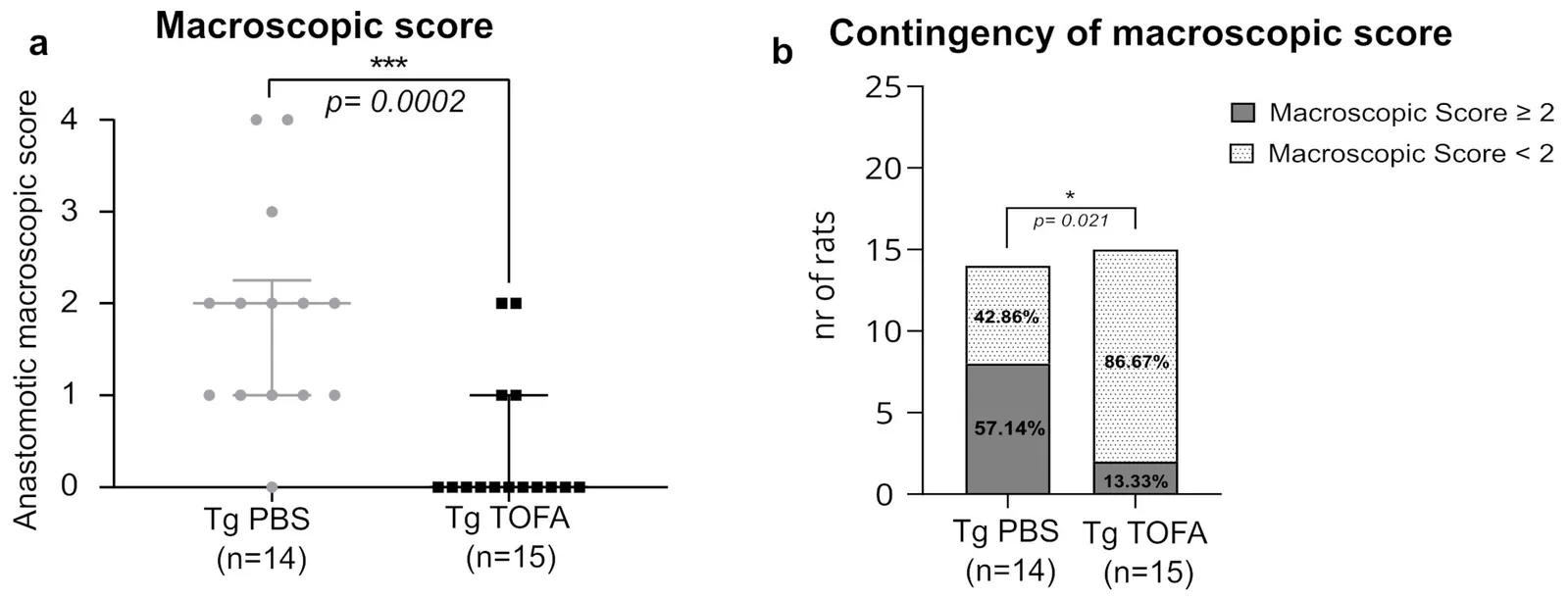
Anastomotic macroscopic lesions and POR at W18. (**a**) Scatter plots for anastomotic macroscopic scores assigned at W18 to Tg rats receiving PBS and TOFA. Data are quoted as median scores with IQR and analyzed by the Mann–Whitney test (*** = *p* < 0.001). (**b**) Histogram depicting the frequency distribution of rats with macroscopic score ≥2 or <2 in Tg PBS and Tg TOFA groups. Data are expressed as percentages with 95% CI and analyzed using Fisher’s exact test (* = *p* < 0.05).

### 2.3. Endoscopic Assessment at W15 and Predictive Value for POR at W18

Mucosal inflammation was monitored by endoscopic evaluation of the terminal ileum 3 weeks after ICR (W15), which is a minimally invasive method. All TOFA-treated rats showed mild lesions (score ≤ 6, median 6, IQR: 3–6), whereas all PBS-treated rats exhibited significantly more severe lesions (score ≥ 6, median 9.5, IQR: 9–10.25) (*p* < 0.0001) (Figure 3a). Endoscopic severity at W15 was strongly correlated with the development of macroscopic lesions at W18 (r = 0.7783, *p* < 0.0001) (Figure 3b).

**Figure 3:**
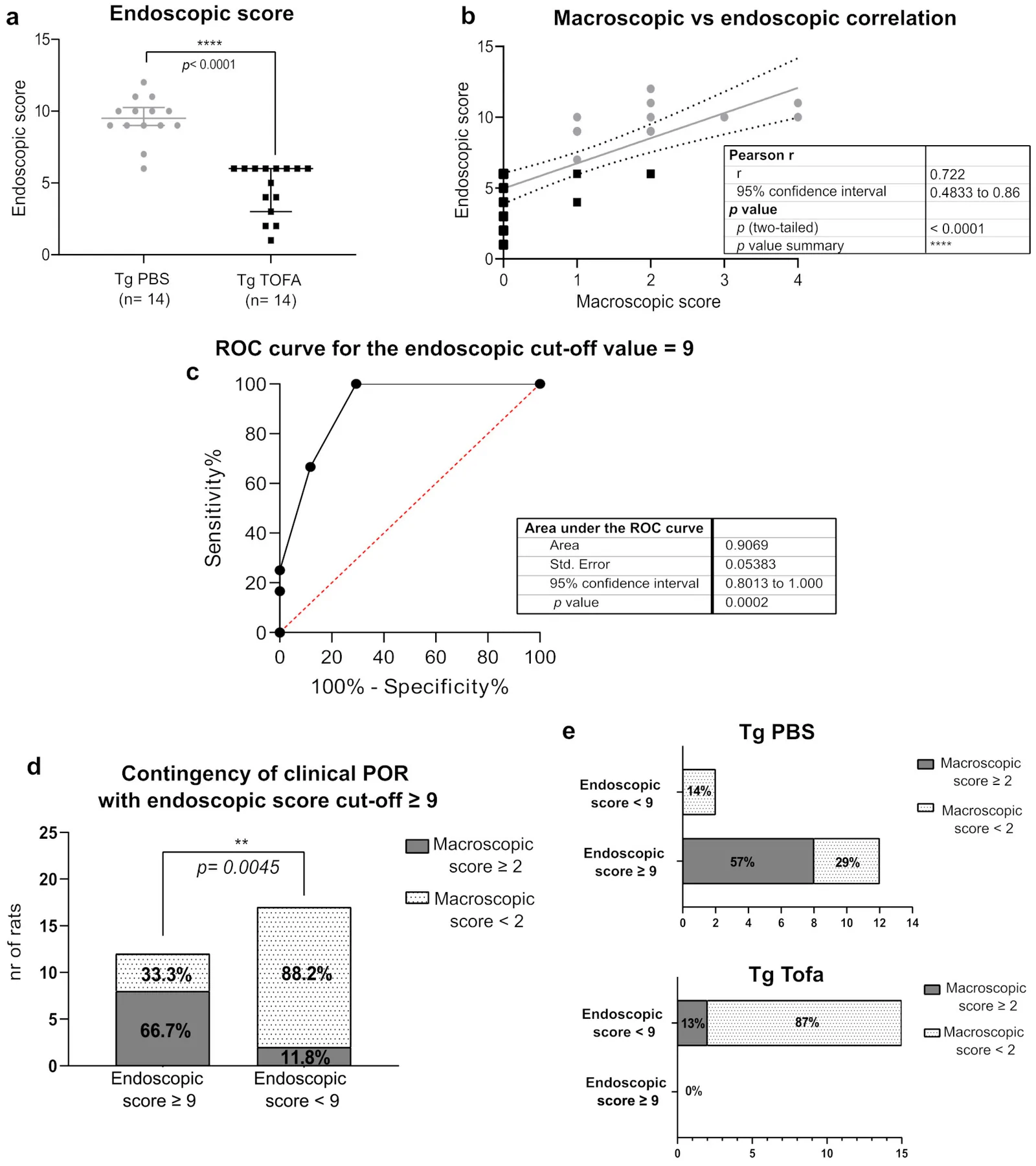
Early endoscopic evaluation. (**a**) Scatter plots for anastomotic endoscopic lesions expressed as median scores with IQR and analyzed by Mann–Whitney test (**** = *p* < 0.0001). (**b**) Correlation and linear regression between the median macroscopic and endoscopic scores of anastomotic lesions in both Tg PBS (grey dots) and Tg TOFA (black squares) rats. Data are analyzed using Spearman’s rank correlation (**** = *p* < 0.0001). (**c**) ROC curve for the endoscopic score (*p* < 0.0001). (**d**,**e**) Frequency distributions of rats in (**d**) both and (**e**) separated groups using macroscopic score ≥ 2 and the endoscopic cut-off score of 9 as variables. Data are expressed as percentages with 95% CI and analyzed by Fisher’s exact test (** = *p* <0.01).

Using a ROC-derived endoscopic cut-off of 9 (AUC: 0.9069 and 95% CI: 80.13% to 100%) (Figure 3c), we estimated that POR occurred in 8/12 rats with high scores (66.67%) and in 2/17 animals with low scores (11.8%) (*p* = 0.0045) (Figure 3d). Significant endoscopic lesions (score ≥ 9) at W15 had a positive predictive value (PPV) of 66.7% (or a negative predictive value (NPV) of 88.24%) with 80% sensitivity (with 95% CI: 44.39% to 97.48%) and 78.95% specificity (with 95% CI: 54.43% to 93.95%) and a global prevalence of 34.48% (10/29 rats) (with 95% CI: 17.94% to 54.33%). According to treatment, 85% (12/14) of PBS-treated rats developed significant endoscopic lesions, leading to POR at W18 in 57% of animals (8/14). In contrast, none of the TOFA-receiving rats developed lesions ≥9, suggesting a protective effect of TOFA against intestinal recurrence potentially obtained within five weeks of treatment.

### 2.4. Histologic Anastomotic Lesions at W18

Histological analysis of the anastomotic area evidenced the presence of mucosal erosion and inflammatory infiltration (referable to a score ≥ 2) in 12/14 rats receiving PBS at W18. In 4 of these rats, histologic lesions extended over 30% of the analyzed tissue (score 3). The other 5 rats showed the presence of ulcerations (score 4), that in one of these rats interested70% of the whole intestinal section (score 6) (Figure 4a). The median histologic score for the Tg PBS group was 3 (IQR: 2–4). The daily TOFA administration significantly reduced this score to 2 (IQR: 1–3, *p* = 0.03) (Figure 4b). We also quantified the number of inflammatory cells infiltrating the ileal lamina propria, which was significantly decreased in Tg TOFA rats, compared to untreated animals (68 × 10^3^ cells/µm^2^, IQR: 46.15 × 10^3^–88.4 × 10^3^ vs. 133.3 × 10^3^ cells/µm^2^, IQR: 59.5 × 10^3^–178 × 10^3^; *p* = 0.034) (Figure 4c). The number of infiltrated cells significantly correlated with the histologic score (r = 0.732; *p* <0.0001) (Figure 4d).

**Figure 4:**
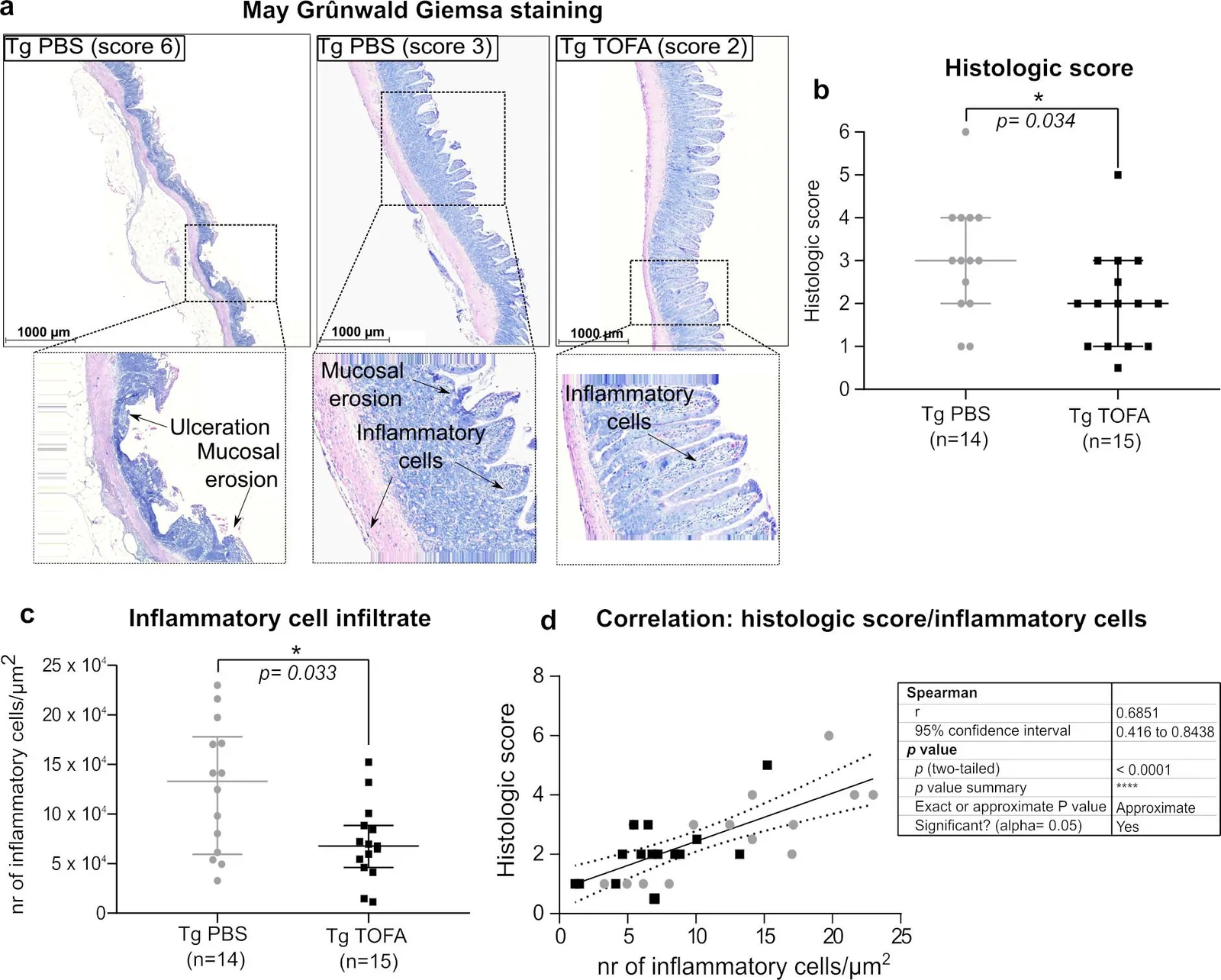
Histologic evaluation of the anastomotic area. (**a**) Representative microphotographs of ileal samples recovered at 2 cm from the anastomosis at W18 stained with May-Grünwald Giemsa coloration and scanned at 20× magnification. (**b**) Scatter plots for the histologic score, expressed as median value with IQR for both groups and analyzed by Mann–Whitney test (* = *p* < 0.05). (**c**) Scatter plots depicting the ImageJ-assisted quantification of the number of inflammatory cells infiltrate/µm^2^, expressed as median value with IQR for both groups and analyzed by Mann–Whitney test (* = *p* <0.05). (**d**) Correlation curve between histologic score and inflammatory infiltrate assessed using Pearson’s rank analysis (**** = *p* < 0.0001).

### 2.5. STAT Phosphorylation in Ileal Samples at W18

We investigated STAT3 phosphorylation by Western blot in ileal samples collected at W18. TOFA-treated Tg rats exhibited a significant reduction in the pSTAT3/STAT3 ratio, with a median fold change of 0.5 (IQR: 0.06–0.84) versus 1.16 (IQR: 0.72–1.62) in the PBS group (*p* = 0.0023) (Figure 5).

**Figure 5:**
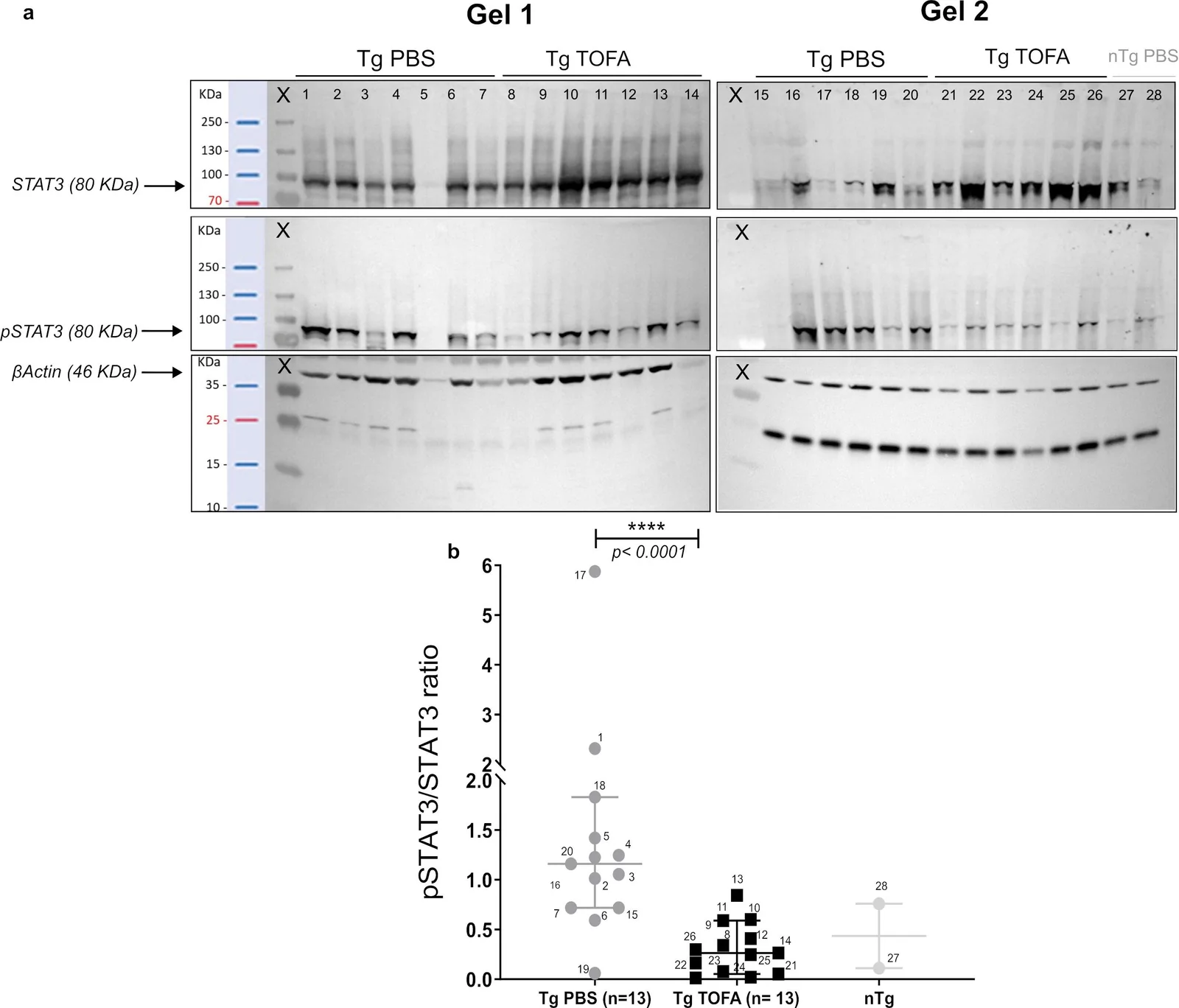
Western blot analysis of STAT3 phosphorylation in ileal samples at W18. (**a**,**b**) Representative Western blot analyses of (**a**) total STAT3 and phosphorylated STAT3 (pSTAT3) levels. Gel 1 included samples from 7 Tg rats receiving PBS and 7 Tg rats receiving TOFA. Gel 2 included samples from 6 Tg rats receiving PBS, 6 Tg rats receiving TOFA, and 2 nTg rats receiving PBS from previous studies that are otherwise not included in the present manuscript. The nTg rats were included as reference controls and were excluded from the statistical analysis. (**b**) Scatter plots depicting the pSTAT3/STAT3 ratio in each Tg group, expressed as median value with IQR for both groups and analyzed by Mann–Whitney test (**** = *p* < 0.0001). X indicates the molecular weight marker lane for each gel. Numbers in the scatter plots correspond to the individual lanes of the Western blots for each experimental group shown in (**a**).

### 2.6. Localization and Quantification of pSTAT3-Positive Cells in Transparietal Anastomotic Samples

We evaluated the number and localization of pSTAT3-positive cells in transparietal anastomotic samples by immunohistochemistry. Comparison between tissues collected at surgery and sacrifice demonstrated a significant increase in the median percentage of positive staining in the total area from 0.036 (IQR: 0.026–0.042) at W12 to 0.061 (IQR: 0.044–0.122) at W18 (*p* = 0.0002) in Tg PBS rats the majority of these cells were mainly localized in the lamina propria, suggesting the activation of the JAK/STAT pathway prevailing in immune cells. This increase was significantly reduced by TOFA to 0.02 (IQR: 0.016–0.03) at W18 (*p* < 0.0001). Pairwise comparison in the Tg TOFA group also demonstrated that the preventive administration of TOFA did not induce any significant increase in the number of pSTAT3-positive cells between the surgery and sacrifice (Figure 6).

**Figure 6:**
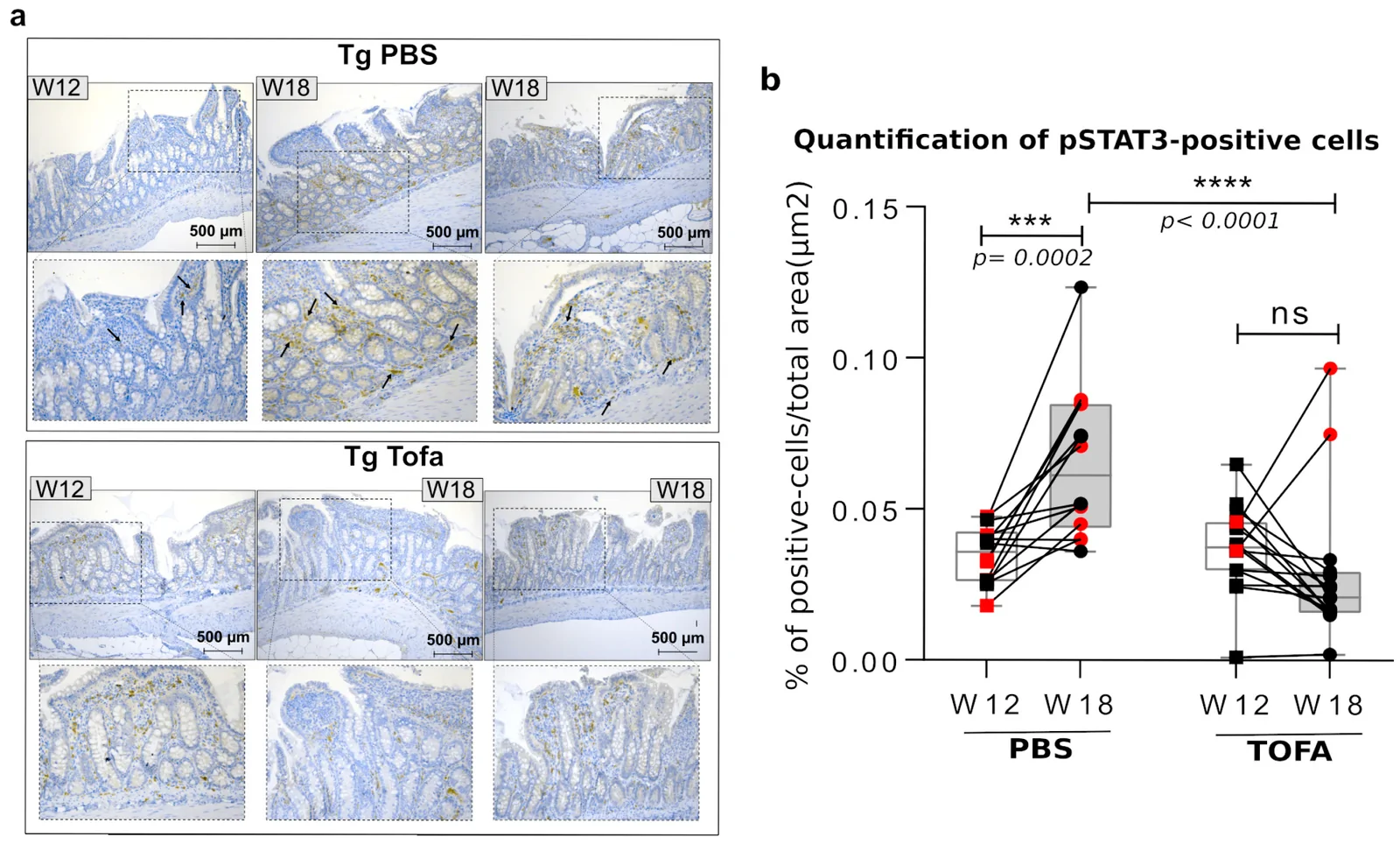
Immunohistochemical analysis for pSTAT3 expression in the anastomotic area. (**a**) Representative microphotographs of immunohistochemistry for pSTAT3 in samples recovered at 2 cm from the anastomosis at W12 and W18 and scanned at 20× magnification, with black arrows indicating pSTAT3-positive cells. (**b**) Scatter plots depicting the ImageJ-assisted quantification of the number of pSTAT3-positive cells/total area (µm^2^), with red dots indicating rats developing macroscopic lesions (score ≥ 2) at W18. Data are expressed as median values with IQR for both groups and analyzed with a Two-way ANOVA with Tukey’s correction for multiple pairwise comparisons (*** = *p* < 0.001) and Sidak’s correction for comparison between unmatched data (**** = *p* < 0.001).

### 2.7. mRNA and Protein Expression Levels of Pro-Inflammatory Cytokines in the Anastomotic Area

Gene expression levels of pro-inflammatory cytokines in Tg PBS rats were significantly increased in the anastomotic area, from surgery (W12) to sacrifice (W18). Comparison between W12 and W18 showed significant increases in the median relative expression of *Tnf* [2 × 10^−3^, IQR: (1.27–2.57) × 10^−3^ vs. 1.2 × 10^−3^, IQR: (0.6803–1.82) × 10^−3^, *p* = 0.028], *Ifn* [0.2 ×10^−3^, IQR: (0.08–0.6) × 10^−3^ vs. 0.066 × 10^−3^, IQR: (0.02–0.12) × 10^−3^, *p* = 0.037], *Il17* [0.38 × 10^−3^, IQR: (0.3–0.77) × 10^−3^ vs. 0.094 ×10^−3^, IQR: (0.04–0.27) × 10^−3^, *p* = 0.033], *Il23* [0.1 × 10^−3^, IQR: (0.07–0.48) × 10^−3^ vs. 0.076 × 10^−3^, IQR: (0.04–0.17) × 10^−3^, *p* = 0.005] and *Il6* [0.07 × 10^−3^, IQR: (0.05–0.09) × 10^−3^ vs. 0.027 × 10^−3^, IQR: (0.012–0.05) × 10^−3^, *p* <0.0001], with a not significant trend of *Il1b* [5.41 × 10^−3^, IQR: (1.17–2.8) × 10^−3^ vs. 3.94 ×10^−3^, IQR: (2.44–6.28) × 10^−3^] (Figure 7a–f). The daily TOFA administration markedly reduced the upregulation of *Tnf* (by 62%, *p* = 0.0003), *Ifn* (by 58%, *p* = 0.03), *Il17* (by 78%, *p* = 0.01), *Il23* (by 21%, *p* = 0.0075), *Il6* (by 68%, *p* <0.0001), *Il1b* (by 59%, *p* = 0.0014) at W18, compared to Tg PBS group (Figure 7a–f). IL-17 protein levels were also measured in whole frozen spleen, mesenteric lymph nodes and ileum at W18 (Figure 7g). As expected in the HLA-B27 Tg rat, IL-17 concentrations were higher in splenic lysates of both groups than in mesenteric lymph nodes or ileum. However, compared to untreated rats, TOFA significantly reduced IL-17 levels in the spleen [1.3 × 10^−7^, IQR: (0.8–2.22) × 10^−7^ vs. 2.23 × 10^−7^, IQR: (1.5–3.12) × 10^−7^, *p* = 0.014 ], as well as in the mesenteric lymph nodes [0.077 × 10^−7^, IQR: (0.042–0.16) × 10^−7^ vs. 0.8 × 10^−7^, IQR: (0.5–0.87) × 10^−7^, *p* <0.0001] and ileum [0.22 × 10^−7^, IQR: (0.3–0.29) × 10^−7^ vs. 0.5 × 10^−7^, IQR: (0.28–0.7) × 10^−7^, *p* = 0.006] (Figure 7g).

**Figure 7:**
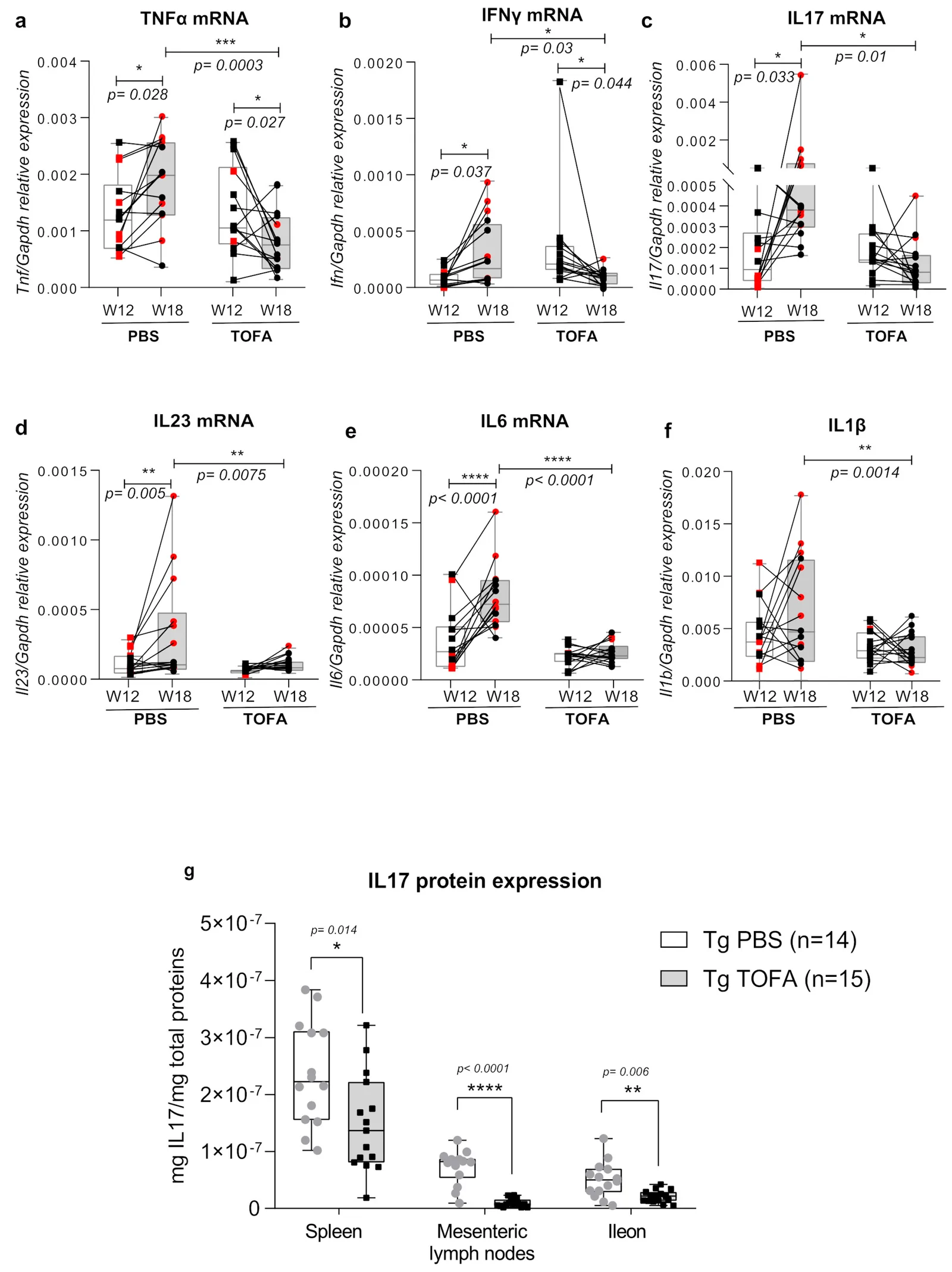
Cytokine profile before and after the ICR. (**a**–**f**) Box-and-whisker plots displaying gene expression levels of pro-inflammatory cytokines (*Tnf*, *Ifn*, *Il17*, *Il23*, *Il6* and *Il1b*) in the anastomotic area of Tg PBS and Tg TOFA rats, quantified at W12 and W18 by qRT-PCR. Red dots indicate rats developing macroscopic lesions (score ≥ 2) at W18. Data were expressed as the median relative expressions to the housekeeping gene *Gapdh* and analyzed with a Two-way ANOVA with Tukey’s correction for multiple pairwise comparisons (* = *p* < 0.05, ** = *p* < 0.01, **** = *p* < 0.0001) and Sidak’s correction for comparison between unmatched data (* = *p* < 0.05, ** = *p* < 0.01, *** = *p* < 0.001, **** = *p* < 0.0001). (**g**) Box and whiskers plots depicting the IL-17 concentration (mg/mg total proteins) levels quantified by ELISA on frozen lysates of the spleen, mesenteric lymph nodes and ileal samples recovered at the sacrifice (W18) from Tg PBS and Tg TOFA groups. Data were quoted as median IL-17 concentration (mg/mg of total protein) and analyzed using the Mann–Whitney test (* = *p* < 0.05; ** = *p* < 0.01; **** = *p* < 0.0001).

## 3. Discussion

Developing effective therapies to prevent post-operative recurrence (POR) in Crohn’s disease (CD) remains a major challenge, as its underlying pathophysiology is incompletely understood and evidence-based treatment strategies are limited. Preclinical models are therefore essential to elucidate the mechanisms underlying POR and evaluate novel therapeutic approaches. To address this need, our group developed a robust POR model based on ileocecal resection in HLA-B27/human β2-microglobulin transgenic (HLA-B27) rats, which closely recapitulates key features of human inflammatory bowel disease (IBD) and spondylarthritis [19]. The molecular basis of multisystemic inflammation in HLA-B27 rats stems primarily from endoplasmic reticulum stress caused by misfolded HLA-B27 heavy chains, which activate the unfolded protein response, particularly in macrophages [20]. This immune dysregulation promotes IL-23 overproduction and the expansion of a Th17-type immune response, leading to increased IL-17 expression alongside other pro-inflammatory cytokines, including IL-1β, IL-6, TNF-α, IL-12, MIP-2 and IFN-γ, which are primarily associated with a Th1-type inflammatory profile [20,21,22,23]. In this immunological context, rats from our colony develop spontaneous colitis from 10 weeks of age. Following ICR in 12-week-old rats, macroscopic examination of the peri-anastomotic area revealed ileal edema, ulcerations, and stenosis 6 weeks post-surgery [24]. The extent of these lesions and progression of inflammation were quantified using a tailored scoring system ranging from 0 to 4. Within this framework, a score ≥2 (corresponding to ulcerations involving more than 20% of the anastomotic area) was used to define the clinical POR.

This immune dysregulation promotes IL-23 overproduction and the expansion of a Th17-type immune response, leading to increased IL-17 expression alongside other pro-inflammatory cytokines, including IL-1, IL-6, TNF-α, IL-12, MIP-2, and IFN-γ, which are primarily associated with a Th1-type inflammatory profile [20,21,22,23]. In this immunological context, rats from our colony develop spontaneous colitis from 10 weeks of age. Following ileocolic resection (ICR) in 12-week-old rats, macroscopic examination of the peri-anastomotic area revealed ileal edema, ulcerations, and stenosis at 6 weeks post-surgery [24]. Lesion severity and inflammatory progression were assessed using a dedicated scoring system ranging from 0 to 4. A score ≥2, corresponding to ulcerations affecting more than 20% of the anastomotic area, was used to define clinical postoperative recurrence (POR).

Identifying patients at high risk of POR is crucial for managing CD-associated POR. The POCER study, a randomized trial of 85 patients, found that ulcerations observed 6 months after ICR via endoscopy were more predictive of clinical POR progression than scores like the Crohn’s Disease Endoscopic Index of Severity (CDEIS), the Simple Endoscopic Score for Crohn’s Disease (SES-CD), and the Rutgeerts’ score [25]. Ulceration depth and extension were used to create the POCER index, which helps to predict POR progression and severity. While the validity of early endoscopic evaluations in humans is still debated, our preclinical study in HLA-B27 rats found a strong correlation between endoscopic scores at W15 and macroscopic scores at W18. Specifically, an endoscopic score of 9 at W15 showed high specificity and sensitivity, with a positive predictive value of 66.67% for developing macroscopic lesions associated with a score ≥ 2, highlighting the potential of early endoscopic evaluation as a minimally invasive monitoring tool for predicting POR.

A gene set enrichment analysis (GSEA) recently performed on a cohort of 45 CD patients identified IL-6/Janus kinases (JAK)/Signal Transducers and Activators of Transcription (STAT) signaling as a specific and highly expressed hallmark pathway in patients with clinical recurrence [26]. As expected, in this preclinical study, we observed a significant increase in STAT3 phosphorylation in the ileum associated with ICR. Additionally, we examined which cell types predominantly expressed pSTAT3. The presence of pSTAT3 in the ileal *lamina propria* of untreated Tg rats at W18 suggested a specific activation of the JAK/STAT pathway in immune cells in response to ICR.

The JAK/STAT pathway, especially STAT3, is crucial in Th cell differentiation in IBD. STAT3 is linked to IL-23-induced IL-17 production and indirectly influences TNFα expression [27]. A recent study by Ngollo et al. highlighted abnormal STAT3 upregulation in the intestinal immune and epithelial cells of patients with early severe endoscopic recurrence, identifying JAK/STAT activation as a key predictor of recurrence. They also discovered that the TNF, IL-17/IL-23 axis, and IFN cytokines form a specific molecular signature of POR [11]. This pro-inflammatory cytokine network, characterized by mixed Th-1/Th-17 profiles, regulates immune responses and inflammation in IBD, particularly in CD [28]. These findings are consistent with the GSEA recently performed by Chen et al., which identified other hallmark inflammatory pathways, including IL-1β and TNFα/NF-κB, as being associated with POR development, although their combined scores were consistently lower than those of the IL-6/JAK/STAT3 pathway [26].

In agreement with all these findings, we demonstrated that in HLA-B27 rats undergoing ICR, the cytokine-associated POR network expanded significantly between surgery and sacrifice, with increased production of TNFα, IL-17, IL-23, and IFN-γ in rats with macroscopic lesions corresponding to a score ≥ 2 (Supplementary Figure S1). The strong correlation between the macroscopic score and the cytokine network profile in our model further supports the use of a macroscopic score ≥ 2 as an indicator of clinical POR. Based on this evidence, we investigated whether pharmacological inhibition of the prominently activated JAK/STAT pathway using the pan-JAK inhibitor tofacitinib (TOFA; CP-690,550) could exert effects beyond the reduction of STAT3 phosphorylation by modulating a broader inflammatory response relevant to POR. TOFA is a small-molecule inhibitor that targets the ATP-binding site of JAK1 and JAK3, thereby preventing STAT3 and phosphorylation and modulating signaling downstream of multiple type I and type II cytokine receptors [15].

This study demonstrated that in ICR-receiving HLA-B27 rats treated with 15 mg/Kg TOFA daily by oral gavage, STAT3 phosphorylation decreased together with the number of pSTAT3-positive cells in the neo-terminal ileum, supporting effective inhibition of JAK/STAT3 signaling in vivo. This molecular effect was associated with a significant improvement in postoperative recurrence, as evidenced by a marked reduction in the overall macroscopic score. Notably, only 2 of 15 TOFA-treated rats developed extended ulceration (macroscopic score ≥ 2) (Figure 4). This improvement was also confirmed by a significantly lower early endoscopic score at W15 in rats treated with TOFA. Additionally, TOFA decreased mucosal inflammation, as evidenced by lower histologic scores and reduced inflammatory cell infiltrates compared with untreated rats. In addition, our study showed that tofacitinib downregulated gene expression of key cytokines associated with JAK/STAT3 signaling, such as IL-6, and with other inflammatory pathways, such as TNFα, IL1β and the IL-23/IL-17 axis, six weeks after surgery. The decrease in IL-6 by TOFA was also consistent with previous studies demonstrating its ability to suppress IL-6 expression in rheumatoid arthritis [29]. Western blot analysis revealed the persistence of a residual pSTAT3 signal. Our experimental design does not allow us to determine whether TOFA induced partial rather than complete inhibition of STAT3 signaling, and therefore we cannot establish whether this residual STAT3 activation retains a specific biological function. Nevertheless, the persistence of pSTAT3 does not necessarily contradict the observed therapeutic effects of TOFA and may indicate that JAK inhibition acts through partial modulation of the broader inflammatory network rather than complete suppression of STAT3 signaling. This interpretation is consistent with the modulation of multiple inflammatory mediators observed in our model. Another hypothesis is that TOFA could exert modulation of additional STAT-dependent pathways, including STAT1 and STAT6. Although STAT1 and STAT6 phosphorylation were not directly assessed in the present study, our findings provide limited indirect evidence regarding the involvement of these pathways. Ileal IFN-γ expression, which is associated with STAT1 signaling, was transiently reduced following TOFA treatment. Regarding STAT6, although JAK inhibition could theoretically affect IL-4- and IL-13-mediated signaling, neither cytokine was detectable in ileal tissue at baseline in either the PBS- or TOFA-treated groups. Thus, our data do not provide evidence of IL-4/IL-13–STAT6 axis activation in this model. Further studies directly assessing STAT1 and STAT6 phosphorylation would be required to clarify their respective contributions to postoperative recurrence and the effects of TOFA. Lastly, although no marked changes in the gut microbiota of HLA-B27 rats were detected following TOFA treatment, particularly concerning overall microbial diversity, as previously reported [30], we cannot exclude an indirect contribution of changes in the symbiont–pathobiont balance to the anti-inflammatory effects observed.

Although mechanistic novelties require further investigation to increase the translational relevance of our findings, and although our experimental design differs from the clinical setting of postoperative prophylaxis, in which treatment is generally initiated after surgical resection, the present findings provide a proof-of-concept for the efficacy of pharmacological JAK/STAT inhibition in preventing POR.

Regarding the safety profile of prolonged tofacitinib exposure, the absence of mortality, together with the stability of body weight and DAI score, provides supportive evidence of treatment tolerability in the present study. However, these parameters do not constitute a formal safety assessment. Dedicated studies assessing hematological parameters, organ toxicity, antimicrobial immunity and wound/anastomotic healing would be required to fully characterize the safety and translational implications of prolonged tofacitinib exposure in the postoperative setting.

Nevertheless, these findings should also be interpreted in the context of the specific pharmacological properties of tofacitinib. In the present study, tofacitinib was used primarily as a pharmacological tool to investigate the contribution of JAK/STAT signaling to postoperative recurrence, rather than to establish a class-wide effect of JAK inhibition. Other JAK inhibitors with distinct selectivity profiles, such as the JAK1-selective inhibitor upadacitinib, may modulate downstream signaling differently. Whether such pharmacological differences translate into distinct effects on postoperative Crohn’s disease recurrence remains an important question for future studies.

## 4. Materials and Methods

### 4.1. Animals

HLA-B27 transgenic rats (Tg) were provided by Professor M. Breban (Cochin Institute, INSERM U1016, Paris, France). Rats were maintained in a specific pathogen-free facility at the Institut Pasteur de Lille (France) under controlled temperature (22 ± 2 °C) and humidity (35% to 75%). Rats were exposed to 12 h light/dark cycles and fed a standard diet with free access to water. Animal experiments were conducted in accordance with European Directive 2010/63/UE, as implemented by French Decree No. 2013-118, and were approved by the regional ethics committee CEEA75. The animal study protocol was also approved by the French government (APAFIS authorization number #17092-2018092818147464, decision date: 6 April 2019).

### 4.2. Tofacitinib

Tofacitinib citrate (Pfizer, Paris, France) (TOFA; CP-690,550) was dissolved in PBS and administered twice daily by oral gavage at the optimal concentration of 7.5 mg/kg (15 mg/Kg/d). The dose of TOFA was defined by previous preclinical models of colitis and corresponds to the human equivalent daily dose for ulcerative colitis, recommended by the Food and Drug Administration (FDA) [31,32].

### 4.3. Experimental Design

Twenty-nine age-matched and sex-mixed Tg rats were included in the study and randomized into: i. Tg PBS group (*n*= 14, 6 males and 8 females), receiving a placebo composed of PBS and ii. Tg TOFA group (*n*= 15, 8 males and 7 females), receiving TOFA, as previously described. The randomization strategy complied with the ARRIVE guidelines and was based on the blocking method, considering treatment as a variable [33]. The sample size was determined based on previous preclinical studies evaluating tofacitinib in experimental models of colitis, which used comparable group sizes and reported significant treatment effects. No formal a priori sample size calculation was performed. The number of animals was also kept to a minimum in accordance with the principles of animal welfare and reduction, while aiming to obtain reliable and interpretable results. To minimize potential allocation bias, the allocation sequence was generated by CD using block randomization, with the numbers of animals assigned to the PBS and TOFA groups balanced within each of the three experimental blocks. Placebo or TOFA was administered by intragastric gavage to conscious animals, with a preventive strategy starting from 10 weeks of life (W10) until W18, with the aim of ensuring adequate JAK/STAT pathway inhibition and drug exposure at the time of surgical injury. At W12, rats from both groups underwent an ileocecal resection (ICR) of 3 cm in length, followed by end-to-end anastomosis. The ICR procedure was blindly performed under general anesthesia with 2% isoflurane. Briefly, after an abdominal incision, the mesenteric vessels were ligated before the digestive section. The small intestine was resected 3 cm from the ileocecal valve and 3 cm from the caecum. The end-to-end anastomosis was performed with single stitches using polypropylene surgical sutures (Prolène 6.0) and the abdominal wall was closed by separately suturing the muscular layer with 2-0 Vicryl absorbable sutures and the cutaneous layer with size 1 Mersuture non-absorbable sutures. Analgesic treatment was provided with daily subcutaneous injections of 0.03 mg/Kg of Buprenorphine at the surgery and then every 12 h for 3 days (Figure 8). Rats were euthanized 6 weeks after surgery. The W18 endpoint was established in our previous studies, which identified this time point as appropriate for distinguishing recurrent intestinal inflammation from transient postoperative inflammation. The timeline diagram and rats’ repartition are illustrated in Figure 1.

**Figure 8:**
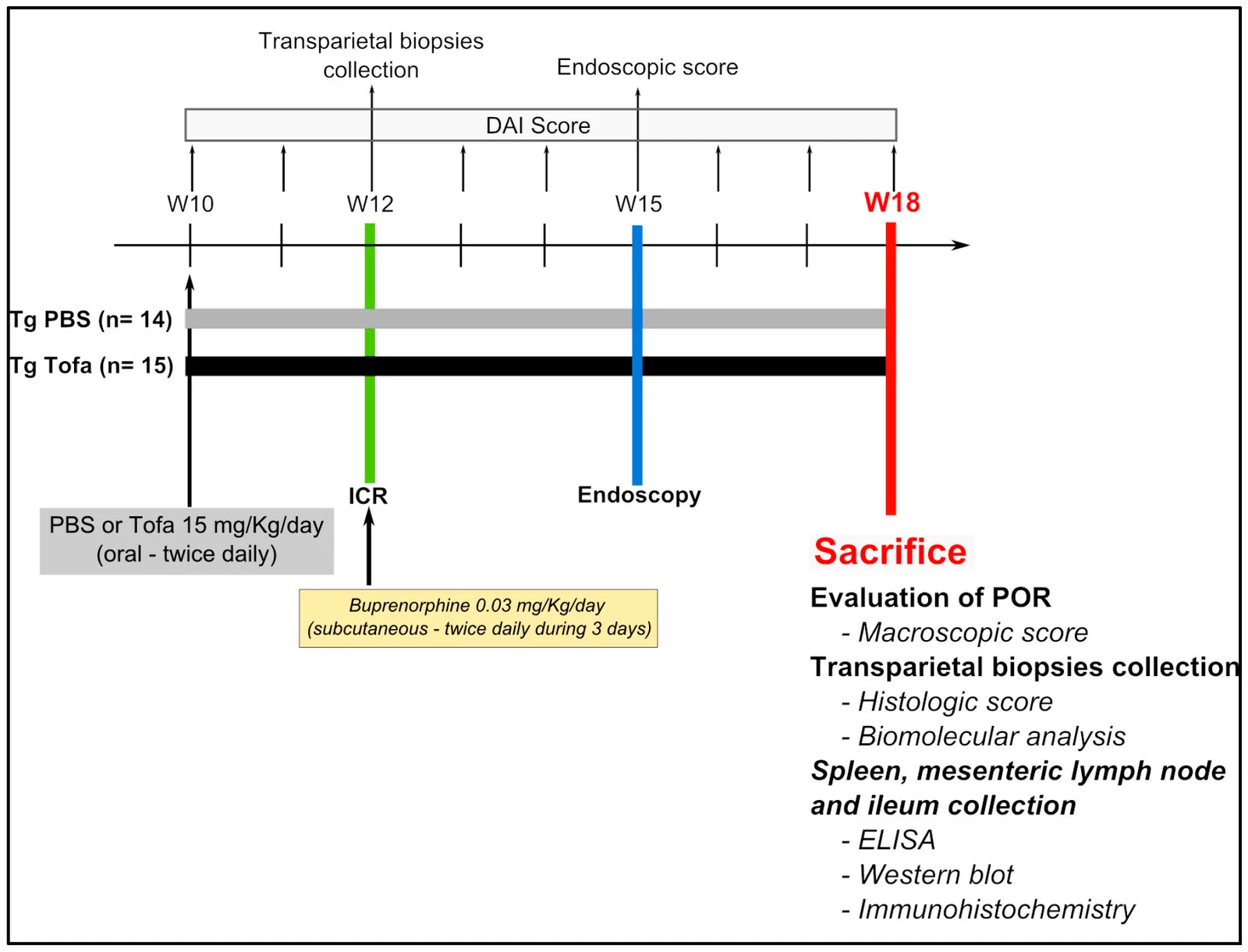
Outline of experimental design.

### 4.4. Evaluation of Clinical Parameters

Body weight changes, diarrhea and macroscopic bloody stools were monitored and scored as in Table 1 once weekly until the rats were euthanized. The sum of each score was calculated as the total disease activity index (DAI) value (ranging from 0 to 5). Body weight data were expressed as the percentage of the baseline body weight measured at W10 (set to 100%). Disease Activity Index (DAI) scores were expressed as the median with interquartile range (IQR).

**Table 1:** Disease Activity Index (DAI) Score (0–5).

Parameters	Findings		
	**0**	**1**	**2**
**Weight loss**	None	<10%	≥10%
**Stool consistency**	Normal	Soft	Liquid
**Blood**	Absence	Macroscopic	n.d.

### 4.5. Evaluation of Endoscopic Scores (0–15) at W15

At W15, rats underwent endoscopic evaluation under anesthesia using a flexible fiberoptic bronchoscope (Pentax FB 8v; PENTAX Medical, Hamburg, Germany), 2.7 mm diameter, 60 cm length) to visualize the terminal ileum [32]. Colon cleaning was performed 24 h before endoscopy by replacing drinking water with a solution containing one Macrogol sachet (Klean Prep, Vidal) and 30 g of sucrose per liter. Anastomotic and ileocolonic inflammation were assessed using the Murine Endoscopic Index of Colitis Severity (MEICS), which scores five parameters (0–3 each), as shown in Figure 9 [34,35]. Endoscopic scores were accepted when an inter-observer coefficient of variation was <15%.

**Figure 9:**
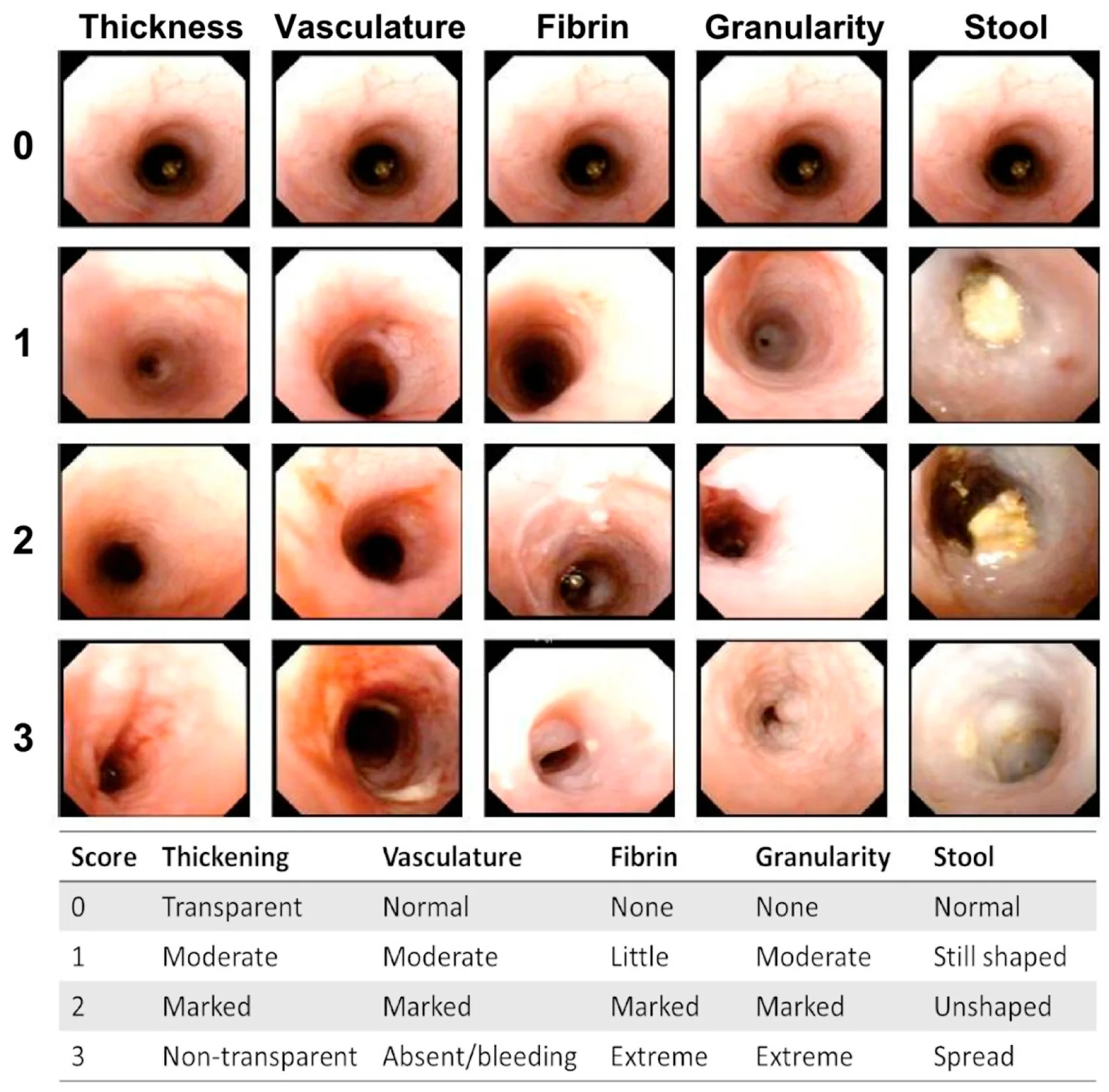
Endoscopic images and relative MEICS score.

### 4.6. Macroscopic Evaluation and Definition of Post-Operative Recurrence (POR) at W18

Six weeks after surgery (W18), rats were euthanized and the intestine was macroscopically blindly evaluated and scored at the anastomotic level (± 2 cm) using a macroscopic grading scale adapted from Rutgeert’s score, ranging from 0 to 4 (Figure 10) [3], as previously published and summarized in Figure 10 [24]. Scores were considered reliable when an inter-observer coefficient of variation was <15%. By analogy with endoscopic recurrence in patients with CD [34], POR was defined as a macroscopic score ≥2, corresponding to the presence of ulcerations ± stenosis.

**Figure 10:**
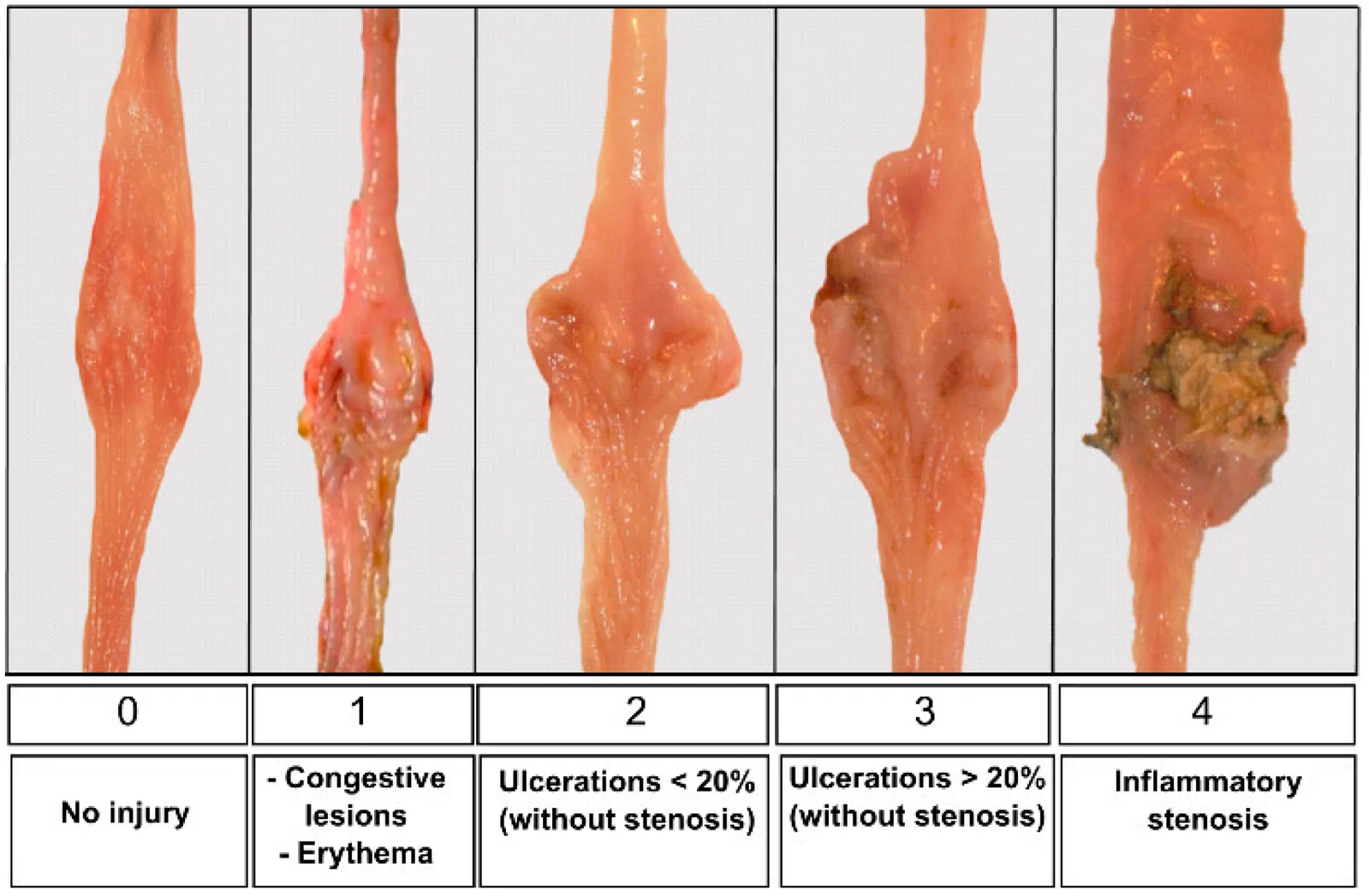
Anastomotic macroscopic score (0–4).

### 4.7. Histologic Evaluation of Anastomotic Lesions

Transparietal anastomotic biopsies were collected during surgery (W12) and sacrifice (W18). Paraffin-embedded tissues were rehydrated with decreasing concentrations of ethanol (from 96% to 70%) and 4 μm serial sections incubated with May-Grünwald (15 min) and Giemsa solutions (40 min) at 37 °C. The histological score (ranging from 0 to 6) was established using the adapted Geboes’ score (Table 2) [36]. The inflammatory infiltrate was quantified using an automated thresholding method in ImageJ 1.54t software. Anastomotic histologic scores were considered reliable when an inter-observer coefficient of variation was <15%.

**Table 2:** Histologic score.

Score	Histologic lesions
**0**	None
**1**	Inflammatory infiltrate and mucosal erosions < 30% of the section
**2**	30% < inflammatory infiltrate and mucosal erosions < 70% of the section
**3**	Inflammatory infiltrate and mucosal erosions > 70% of the section
**4**	Mucosal ulceration < 30% of the section
**5**	30% < mucosal ulceration < 70% of the section
**6**	Mucosal ulceration > 70% of the section

### 4.8. Western Blotting for STAT3 Phosphorylation

STAT3 phosphorylation was assessed in ileal samples from 15 randomly chosen Tg rats (Tg PBS *n* = 7 and Tg TOFA *n* = 8). Total proteins were extracted using RIPA buffer (Sigma-Aldrich, Saint-Quentin-Fallavier, France), separated by SDS-PAGE electrophoresis using Novex 4–12% Tris-Glycine Mini Gels, 15-well WedgeWell™ (ThermoFisher Scientific, Illkirch-Graffenstaden, France) and then transferred onto nitrocellulose membranes using the iBlot 2 Dry Blotting System (ThermoFisher Scientific). Blots were probed with specific antibodies against phospho-STAT3 and STAT3 (Cell Signaling, Ozyme, Saint-Cyr-L’École, France) at a dilution of 1:1000 (overnight at 4 °C) and β-actin (Sigma Aldrich) at a dilution of 1:10,000 (2 h at room temperature) followed by HRP-conjugated secondary antibodies (anti-rabbit or anti-mouse; Sigma Aldrich) at a dilution of 1:2000. Detection was performed using the chemiluminescent substrate Immobilon ECL Ultra Western HRP Substrate (Sigma Aldrich). The signal was acquired using the automatic iBright CL1500 Imaging System (Thermo Fisher Scientific), and the intensity of the bands was quantified using a computer-assisted densitometer in ImageJ software and normalized to β-actin.

### 4.9. Immunohistochemistry

Paraffin-embedded cut ileal sections (4 µm) were rehydrated with decreasing ethanol concentrations (from 96% to 70%), incubated overnight at 4 °C with a specific anti-pSTAT3 antibody (Cell Signaling) and detected using a streptavidin-biotin-peroxidase-conjugated system (Agilent Technologies, Ile-de-France, France) followed by incubation with 3,3-diaminobenzidine (DAB) (Sigma-Aldrich). Finally, after counterstaining with Mayer’s Hematoxylin, sections were examined under a photomicroscope and the number of pSTAT3-positive cells was quantified using ImageJ software.

### 4.10. Quantitative Real-Time (qRT-PCR)

Total RNA was extracted from biopsies of the anastomotic areas using the Nucleospin RNA kit (Macherey-Nagel, Hoerdt, France). A total of 1 µg of RNA was reverse-transcribed into cDNA with the High-Capacity cDNA Reverse Transcription Kit (Thermo Fisher Scientific, Illkirch-Graffenstaden, France). The gene expression levels of inflammatory cytokines such as TNF-α, IL1β, IL6, IL17, IL23, and IFNγ were then measured by qRT-PCR on a StepOne Real-Time PCR System using a dsDNA detector and SYBR Green I chemistry (LightCycler FastStart DNA Master SYBR Green I kit; Thermo Fisher Scientific). Primer sequences are listed in Supplementary Table S1. Relative gene expression was calculated using the formula E = 2^−ΔCt^, where ΔCt is the difference between the critical threshold cycle (Ct) value for each target gene and the Ct of GAPDH. (220–350 nm).

### 4.11. Enzyme-Linked Immunosorbent Assay (ELISA)

The concentration of IL-17/IL-17A was measured in the spleen, mesenteric lymph node and ileum homogenates using an ELISA kit (Bio-Techne), according to the manufacturer’s protocol.

### 4.12. Statistical Analysis

All data were expressed as median (IQR), except the percentage of POR established by macroscopic evaluation, which was presented as mean ± SEM. Body weight variation, DAI score, quantification of pSTAT3-positive cells on immunostained ileal tissues, and cytokine mRNA expression levels were analyzed with a two-way repeated-measures ANOVA’s test with treatment as the between-subject factor and time as the within-subject factor. Measurements obtained at W12 and W18 from the same animal were therefore treated as repeated measurements. Multiple comparisons were performed using Tukey’s test for comparisons within groups over time and Sidak’s test for comparisons between Tg TOFA and Tg PBS rats, as appropriate. As no data were missing, a repeated-measures ANOVA test was used rather than a mixed-effects model. The macroscopic, endoscopic and histologic scores were analyzed by the Mann–Whitney test, as well as the quantification of inflammatory cell infiltrate and the IL-17 production in the spleen, mesenteric lymph nodes and ileum. The proportion of animals with a macroscopic lesion score ≥2 was compared using Fisher’s exact test. Correlations were assessed using Spearman’s rank correlation coefficient. The sensitivity and specificity of the endoscopic score were computed with a receiver operating characteristic (ROC) curve using the Brown/Wilson method. Referencing an endoscopic cut-off point of 9, the positive predictive value (PPV) and the negative predictive value (NPV) were calculated as follows [37]:

$$PPV=\frac{Sensitivity\times prevalence}{Sensitivity\times prevalence+\left(1-Specificity\right)\times (1-Prevalence)}$$

$$NPV=\frac{specificity\times (1-prevalence)}{\left(1-sensitivity)\times prevalence+specificity\times (1-prevalence\right)}$$

All tests were considered statistically significant if *p* < 0.05. Statistical analysis was performed using GraphPad Prism 8.0.1 (GraphPad Software, San Diego, CA, USA).

## 5. Conclusions

Tofacitinib (TOFA) was introduced in 2018 as a promising oral therapy for the management of moderate-to-severe active ulcerative colitis (UC) [38]. Despite subsequent restrictions related to increased risks of thromboembolic events, cardiovascular complications, and malignancies, TOFA remains approved for UC patients who have failed or are intolerant to TNF inhibitors. In the present study, no major short-term tolerability signal was observed following TOFA administration in this experimental model. However, we observed no mortality and stability of clinical parameters, including body weight and DAI score, in HLA-B27 rats treated with TOFA for 8 weeks. Previously, we also demonstrated that TOFA administration did not alter microbiota diversity in HLA-B27 rats [30]. Together, these findings support that TOFA administration was well tolerated in HLA-B27 rats over the treatment period, consistent with evidence from three clinical trials—one Phase II (NCT00615199), two Phase IIb (NCT01393626, NCT01393899)—and an open-label extension study (NCT01470599) in CD [39]. Although these findings cannot be directly extrapolated to clinical postoperative prophylaxis and do not establish a class-wide effect of JAK inhibitors, the reduction in STAT3 phosphorylation observed alongside decreased inflammatory cytokine expression and attenuated intestinal lesions in the HLA-B27 ICR model provides further insight into the inflammatory mechanisms underlying POR. These results support further preclinical investigation of JAK inhibition as a potential strategy for preventing POR in patients with CD.

## Data Availability

The data presented in this study are available on request from the corresponding author.

## Supplementary Materials

The following supporting information can be downloaded at: https://www.mdpi.com/article/10.3390/ijms27219487/s1.

## Abbreviations

The following abbreviations are used in this manuscript:

CD	Crohn’s Disease
POR	Post-operative recurrence
ICR	Ileocecal resection
TNFα	Tumor necrosis factor α
IL	Interleukin
IFN	Interferon
JAK	Janus kinase
STAT	Signal Transducers and Activators of Transcription
HLA-B27	HLA-B27/human β2-microglobulin transgenic rat
Tg	Transgenic
PBS	Phosphate-buffered saline
TOFA	Tofacitinib
IQR	Interquartile range
DAI	Disease Activity Index
PPV	Positive predictive value
NPV	Negative predictive value
CI	Confidence interval
CEEA	Ethical Committee for Animal Experimentation
