# Supplementary material for: Targeting JAK/STAT Signaling Attenuates Postoperative Recurrence in an HLA-B27 Transgenic Rat Model of Crohn’s Disease
Source: Int J Mol Sci. 2026 Oct 24;27(21):9487. doi: 10.3390/ijms27219487 (PMC13762819; doi:10.3390/ijms27219487)
Supplement: Supplementary file 1 [file ijms-27-09487-s001.zip › Supplementary Figure S1.pdf]

Figure S1: Correlation between macroscopic score and the cytokine network profile in HLA-B27 rats undergoing ICR

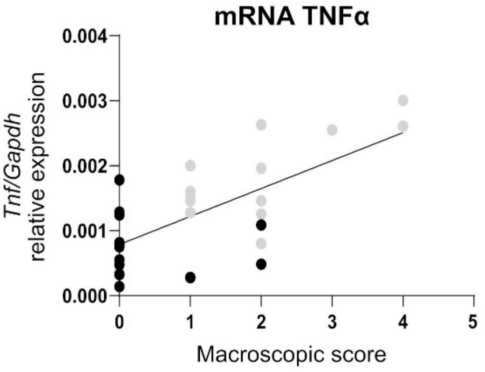

Spearman r	
r	0.5164
95% confidence interval	0.1739 to 0.7475
P value	
P (two-tailed)	0.0041
P value summary	**
Exact or approximate P value?	Approximate
Significant? (alpha = 0.05)	Yes
Number of XY Pairs	29

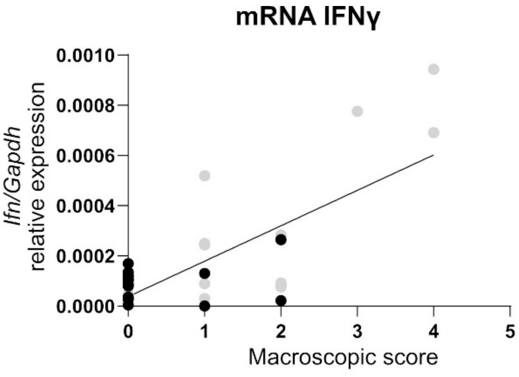

Spearman r	
r	0.3691
95% confidence interval	-0.008365 to 0.6545
P value	
P (two-tailed)	0.0488
P value summary	*
Exact or approximate P value?	Approximate
Significant? (alpha = 0.05)	Yes
Number of XY Pairs	29

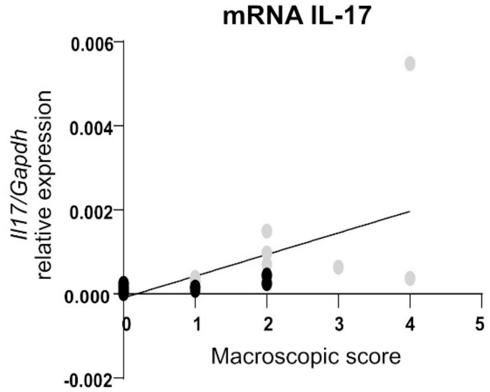

Spearman r	
r	0.8375
95% confidence interval	0.6735 to 0.9230
P value	
P (two-tailed)	<0.0001
P value summary	****
Exact or approximate P value?	Approximate
Significant? (alpha = 0.05)	Yes
Number of XY Pairs	29

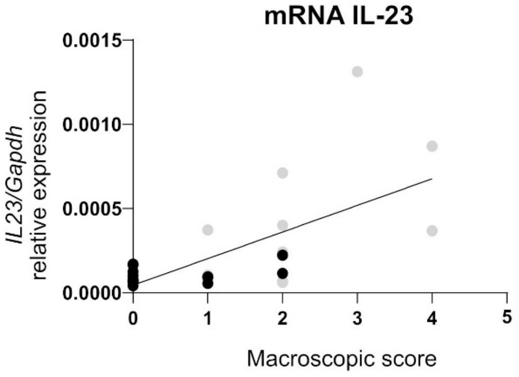

Spearman r	
r	0.5709
95% confidence interval	0.2478 to 0.7797
P value	
P (two-tailed)	0.0012
P value summary	**
Exact or approximate P value?	Approximate
Significant? (alpha = 0.05)	Yes
Number of XY Pairs	29

Figure S1: Correlation between macroscopic score and the ICR-associated cytokine network. Correlation assessed using Spearman's rank and linear regression between the macroscopic and the relative expression of genes coding for TNFα, IFNγ, IL-17 and IL-23 measured at sacrifice on frozen ileal samples collected from Tg rats receiving PBS (grey dots) or TOFA (black dots). ***= p< 0.001; ****= p< 0.0001.
