# Supplementary material for: Targeting JAK/STAT Signaling Attenuates Postoperative Recurrence in an HLA-B27 Transgenic Rat Model of Crohn’s Disease
Source: Int J Mol Sci. 2026 Oct 24;27(21):9487. doi: 10.3390/ijms27219487 (PMC13762819; doi:10.3390/ijms27219487)
Supplement: Supplementary file 1 [file ijms-27-09487-s001.zip › Supplementary Table S1.pdf]

Supplementary Table S1: Primer sequences

Primers	Sequences
Gapdh	Forward: CTG-TTC-TAG-AGA-CAG-CCG-CAT-CT
	Reverse: ACA-CCG-ACC-TTC-ACC-ATC-TTG
Tnf	Forward: GAC-CCT-CAC-ACT-CAG-ATC-ATC-TTC-T
	Reverse: TCC-GCT-TGG-TGG-TTT-GCT-A
Il1b	Forward: CA-ACT-GTC-CCT-CAA-CTC-AA
	Reverse: GT-CAG-CTT-CAA-AGAACA-GG
Il6	Forward: GTTTCTCTCCGCAAGAGACTTC
	Reverse: AGGTTTGCCGAGTAGACCTC
Il17	Forward: ACG-AGG-GAC6AAG-CTC-ATC-CCG
	Reverse: TGG-CCT-CGG-CGT-TTG-GAC-AC
Il23	Forward: CAG-GTT-CCC-ATG-GCT-ACA-GT
	Reverse: TGG-GGT-TTG-TTG-CTT-TTC-TC
Ifn	Forward: GCC-CTC-TCT-GGC-TGT-TAC-TG
	Reverse: CTG-ATG-GCC-TGG-TTG6TCT-TT
